# Characteristics and Detection Rate of SARS-CoV-2 in Alternative Sites and Specimens Pertaining to Dental Practice: An Evidence Summary

**DOI:** 10.3390/jcm10061158

**Published:** 2021-03-10

**Authors:** Sajjad Shirazi, Clark M. Stanford, Lyndon F. Cooper

**Affiliations:** 1 Department of Oral Biology, College of Dentistry, University of Illinois at Chicago, 801 S Paulina St, Chicago, IL 60612, USA; cooperlf@uic.edu; 2 Department of Restorative Dentistry, University of Illinois at Chicago, Chicago, IL 60612, USA; cmstan60@uic.edu

**Keywords:** dentistry, RT-PCR testing, antigen, antibody, saliva, aerosols, body fluids, viral load, epidemiological monitoring, COVID-19

## Abstract

Knowledge about the detection potential and detection rates of severe acute respiratory syndrome coronavirus 2 (SARS-CoV-2) in various body fluids and sites is important for dentists since they, directly or indirectly, deal with many of these fluids/sites in their daily practices. In this study, we attempt to review the latest evidence and meta-analysis studies regarding the detection rate of SARS-CoV-2 in different body specimens and sites as well as the characteristics of these sample. The presence/detection of SARS-CoV-2 viral biomolecules (nucleic acid, antigens, antibody) in different clinical specimens depends greatly on the specimen type and timing of collection. These specimens/sites include nasopharynx, oropharynx, nose, saliva, sputum, bronchoalveolar lavage, stool, urine, ocular fluid, serum, plasma and whole blood. The relative detection rate of SARS-CoV-2 viral biomolecules in each of these specimens/sites is reviewed in detail within the text. The infectious potential of these specimens depends mainly on the time of specimen collection and the presence of live replicating viral particles.

## 1. Introduction

Human viral infection and transmission can occur through multiple routes, including exposure to infected blood, exchange of saliva or aerosols generated from sneezing, coughing or dental procedures, fecal–oral, ingestion of contaminated food and drinks and sexual contact. Common examples of viruses isolated from the oral cavity include coronavirus, norovirus, human immunodeficiency virus (HIV), rotavirus, hepatitis C virus, influenza viruses herpes simplex viruses 1 and 2 and Epstein–Barr virus [1].

The cause of the coronavirus disease 2019 (COVID-19) is the severe acute respiratory syndrome coronavirus 2 (SARS-CoV-2), which is an enveloped, positive-sense single-stranded ribonucleic acid (RNA) virus (+ssRNA). The genome encodes 27 proteins including a number of non-structural proteins, including an RNA-dependent RNA polymerase (RdRP), putative accessory proteins and four structural proteins, named as surface or spike glycoprotein (S), envelope protein (E), membrane protein (M) and nucleocapsid (N) proteins. The virus binds to an angiotensin-converting enzyme 2 (ACE2) receptor through S protein for host cell entry. The virus also has an RNA proofreading mechanism keeping the mutation rate relatively low.

The practice of dentistry necessitates a close contact between the dentist, patient and dental healthcare personnel for patient care and procedure support. In addition, the use of rotary and ultrasonic instruments as well as air–water syringes create aerosols containing particle droplets of water, saliva, blood, microorganisms and other debris. Therefore, the dental setting is a unique environment in the current pandemic since it potentially possesses all transmission risk factors for SARS-CoV-2 virus, as stated by the Centers for Diseases Control and Prevention (CDC). Accordingly, SARS-CoV-2 mainly spreads between people who are in close contact with each other (within 6 feet or 2 m) through respiratory droplets from an infected person. It can linger in aerosols for hours and be spread by people who are not showing symptoms. SARS-CoV-2 can sometimes be spread by airborne transmission within enclosed spaces that have inadequate ventilation within distances more than 6 feet. Contact with contaminated surfaces is another potential transmission route. The infection occurs when the virus is inhaled or deposited on mucous membranes, including that of the nose and mouth [2,3]. Currently, there is no data available to assess the risk of SARS-CoV-2 transmission during dental practice [4,5].

In a recent survey of 849 Italian dentists, a high level of concern for in-office transmission was noted. Dentists perceive needed improvement and change in screening, hygiene and testing patients for SARS-CoV-2 [6]. The Delphi mythology was used among 197 Latin American implant experts to define the importance placed on minimization of disease transmission [7]. When French dental professionals were surveyed early in the pandemic, laboratory-confirmed prevalence of COVID-19 was 1.9% among dentists. Interestingly, practice limited to endodontics (implying general use of rubber dam) was associated with reduced odds of disease [8]. The importance of protecting oral health workers was underscored. A US survey among dentists conducted in June 2020 indicated that 16.6% of participants were tested using respiratory and blood samples, and demonstrated a 0.9% infection rate [9]. A narrative review of Canadian protocols to reduce disease transmission in the dental office included eight different areas involving administrative, physical and procedural controls. The absence of testing was noted as a potential limitation of practice [10].

The presence/detection of SARS-CoV-2 viral biomolecules (nucleic acid, antigens, antibody) in different clinical specimens has been documented based on the type of fluid or material and timing of collection relative to the onset of infection [11]. An infected individual takes an average of 5–6 days (range, 1–14 days) following exposure to develop symptoms (incubation period). The virus may be detectable in the upper respiratory tract 1–3 days before the onset of symptoms, facilitating pre-symptomatic or asymptomatic transmission, but its load is highest around the time of symptom onset, after which it gradually declines [12,13]. Reports recommend that upper respiratory tract samples may have higher infectivity early in the course of the disease (0–5 days), after which the virus starts moving towards the lower parts of the respiratory system. Lower respiratory tract samples may have higher viral load later in the course of disease [14]. 

Current guidance from the World Health Organization (WHO) suggests that the detection of SARS-CoV-2 depends on the testing method, clinical presentation and time since symptom onset [13]. The CDC considers nasopharyngeal, oropharyngeal, nasal and saliva samples to have high viral load and infectivity [15]. In addition, positive detection of SARS-CoV-2 in other clinical samples including sputum, fecal matter, urine, ocular fluid and blood has also been highlighted [16]. 

In this report, we attempt to review the latest evidence regarding the detection rate of SARS-CoV-2 in different body specimens and sites. The knowledge of the detection rate and the infectivity potential of these specimens is essential. This is of particular importance for dentists because they, in their daily practices, directly or indirectly deal with these specimens/sites which might be the port of entry, or replication and transmission site for SARS-CoV-2.

## 2. Detection of COVID-19 in Alternative Samples/Sites

While SARS-CoV-2 can be detected in a wide range of body fluids and compartments, saliva and respiratory samples remain the main choice for diagnostics. Table 1 summarizes the characteristics of the alternative specimens/sites. 

### 2.1. Nasopharynx/Oropharynx

The nasopharynx and oropharynx are main detection sites in early-stage infection of SARS-CoV-2 in both symptomatic and asymptomatic cases. The peak of viral load in nasopharyngeal samples occurs within the first few days after symptom onset. A special type of swabs (flocked swab, synthetic fiber swabs with plastic shafts) is used as the sample collection tool. Calcium alginate swabs or those with wooden shafts are not recommended since they may interfere with nucleic acid amplification tests (NAATs) or contain substances which inactivate the virus [17]. While dependent on the viral load, nasopharyngeal samples are generally more sensitive than oropharyngeal samples. However, the number of days passed since the onset of symptoms and disease stage influence positive testing [13,18,19,20]. The infectivity potential of both specimens has been demonstrated [21].

In a meta-analysis of studies comparing at least two respiratory specimen types (oropharyngeal, nasopharyngeal or sputum), the overall positive detection rate with NAATs in confirmed patients was estimated to be 43% (95% confidence interval (CI): 34–52%) for oropharyngeal swabs and 54% (95% CI: 41–67%) for nasopharyngeal swabs. The estimated percentage of positive tests were 75% (95% CI: 60–88%) between days 0–7, 35% (95% CI: 27–43%) between days 8–14 and 12% (95% CI: 2–25%) after 14 days from symptom onset for oropharyngeal swab sampling. For nasopharyngeal swabs, this figure was 80% (95% CI: 66–91%), 59% (95% CI: 53–64%) and 36% (95% CI: 18–57%) at 0–7, 8–14 and >14 days after symptom onset, respectively [20]. 

A recent meta-analysis of studies comparing paired oropharyngeal and nasopharyngeal samples in confirmed cases found a similar positive detection rate between oropharyngeal and nasopharyngeal swabs (84% (95% CI: 57–100%) vs. 88% (95% CI: 73–98%), respectively) using NAATs. Importantly, there is limited agreement between tests from these sites as the percent of individuals positive for both specimens was only 68% (95% CI: 36–93%) [22]. Nevertheless, combining swabs from both sites has been shown to improve sensitivity and reliability of the results [13]. In addition, a meta-analysis of studies investigating the clinical performance of antigen tests not requiring a separate reading device in confirmed COVID-19 patients revealed a pooled sensitivity of 0.747 (95% CI: 0.673–0.809) for nasopharyngeal or combined oro/nasopharyngeal samples [23].

There are some contraindications for collection of nasopharyngeal samples, including coagulopathy or anticoagulant therapy, and significant nasal septum deviation [24]. Swabs should be placed immediately into a sterile transport tube containing 2–3 mL of either viral transport medium (VTM), Amies transport medium, phosphate-buffered saline, or sterile saline, unless using a test designed to analyze a specimen directly (i.e., without placement in VTM) (Table 1). 

### 2.2. Nasal

Nasal specimen may be obtained with swabs from two anatomical sites, nasal mid-turbinate (deep nasal) and anterior nares, or with nasal wash/aspirate [15]. There is currently no strong evidence regarding SARS-CoV-2 overall positive detection rate in nasal wash compared to other methods. However, Calame et al. [25] compared nasal wash and nasopharyngeal swab sampling and concluded that these methods have comparable clinical and analytical sensitivity.

A meta-analysis of studies comparing paired nasal (either mid-turbinate or anterior nares) and nasopharyngeal samples for NAATs in confirmed cases found that nasal swabs had substantially lower positive detection rate than the nasopharyngeal samples (82% (95% CI: 73–90%) vs. 98% (95% CI: 96–100%), respectively). The percent of individuals positive for both specimens was only 79% (95% CI: 69–88%), suggesting limited agreement. Nasal specimens collected from a single nostril seemed to perform better in comparison to swabs collected from both nares [22]. In addition, studies of only symptomatic patients had a similar positive detection rate for nasal samples as compared to studies of mixed patients. Ultimately, the use of more sensitive assays (limit of detection < 1000 copies/milliliter) for nasal samples resulted in lower positive detection in comparison to assays with limit of detection ≥ 1000 copies/mL. However, this figure was not affected by assay sensitivity in nasopharyngeal samples. This reflects lower viral burden in the mid-turbinate/anterior nares region than the nasopharynx, resulting in lower performance when using highly sensitive assays [22].

A meta-analysis of studies that compared combined oropharyngeal-nasal swabs and nasopharyngeal swabs for NAATs in confirmed cases found an identical positive detection rate (97% (95% CI: 90–100%)) between the two methods. The percent of individuals positive for both specimens was also high (90% (95% CI: 84–96%)) [22]. 

### 2.3. Saliva

The detection of SARS-CoV-2 through oral shedding and especially in saliva has been shown. The infectivity potential of saliva has also been well-demonstrated [26]. SARS-CoV-2 viral load in saliva may be a good indicator of the transmission potential of infected patients, since it is highest during the first week of infection, during which a person is most infectious. 

Saliva has been shown to yield greater detection sensitivity and consistency throughout the course of infection than the nasopharyngeal samples [18,27,28,29,30]. Positive detection rate with NAATs in confirmed cases for saliva samples vary greatly in the literature but is estimated to be higher than 80% [11,31]. A recent meta-analysis of studies comparing paired saliva and nasopharyngeal samples in confirmed cases estimated a positive detection rate of 88% (95% CI: 81–93%) and 94% (95% CI: 90–98%) respectively, with no statistically significant difference. The percent of individuals positive for both the specimens was 79% (95% CI: 71–86%), indicating relatively poor agreement [22]. This study also demonstrated that positive detection rate with NAATs after 7 days from symptom onset was lower compared to ≤7 days (74% (95%CI: 62–85%) vs. 89% (95% CI: 73–99%)), which was also observed for nasopharyngeal swabs in the same patients (91% (95% CI: 82–98%) vs. 99% (95% CI: 90–100%), respectively) [22]. Another meta-analysis estimated an overall diagnostic accuracy of 92.1% (95% CI: 70–98.3%), with sensitivity of 83.9% (95% CI: 77.4–88.8%) and specificity of 96.4% (95% CI: 89.5–98.8) for saliva samples in comparison to nasopharyngeal/oropharyngeal samples in confirmed cases [32]. The sensitivity of saliva was estimated to be 3.4% lower (‒3.4%, 95% CI: ‒9.9–3.1%) than that of nasopharyngeal swabs in another recent meta-analysis [33].

The differences in sensitivity of oral fluids’ evaluations are possibly because of large differences in collection, transport, storage and processing techniques, as well as the evaluation of different testing populations and disease stage. Collection methods included spitting or drooling, coughing or clearing throat, collection with pipet or special sponges and gargling with saline solutions [22,24]. It is likely that a simple drooling technique, with no extra stimulation of saliva secretion, will provide the greatest sensitivity [24]. In addition, many studies have supported the hypothesis of coughing (likely mixed sputum and saliva specimen) or deep throat saliva being better than drool/spit. However, considerable differences have not been revealed [22].

Another difference among studies is sample collection in the morning, or avoidance of eating, drinking, or brushing teeth (30 min to 2 h before specimen collection), which lead to a slightly higher positive detection rate. The variable dilution of saliva prior to processing is another difference among the studies. However, the positivity rate is similar in studies utilizing diluted or undiluted saliva samples. Moreover, studies that directly input the saliva specimen into the amplification assay without any pre-processing showed substantially lower positive detection than those which used a nucleic acid extraction step. Additionally, a positive detection rate in saliva samples was shown to be similar between asymptomatic and symptomatic patients [22]. Ultimately, no substantial difference was detected among studies that used assays with low (<1000 copies/milliliter) or high limit of detection for saliva samples, which demonstrates high viral load in saliva samples [22].

To optimize saliva-based testing and obtain a reliable and sensitive result, a specific, standard and optimized saliva collection and transportation method should be utilized. Also, an optimal solution should be used to collect, transport and store saliva samples. In addition, the RNA isolation and detection protocol should be optimized for saliva, using an appropriate internal control. The use of human DNA is suggested for nasopharyngeal samples but not for saliva samples [24]. The use of saliva samples is quicker, less painful and invasive and allows for higher volume testing, and collection by the patient at home or clinic without posing risks to healthcare providers. Sample includes 1–5 mL of saliva in a sterile, leak-proof screw cap container, with no preservative required (Table 1). The simplicity of saliva-based testing for large populations must be weighed against the reported differences in sensitivity when compared to nasopharyngeal samples.

### 2.4. Sputum

Sputum is mucus produced in the respiratory tract (the trachea and bronchi) and is collected by coughing up deeply and spitting out directly into a sterile, leak-proof, collection cup. It is indicated later in the course of the COVID-19 disease or in patients with a negative upper respiratory sample result while there is a strong clinical suspicion of COVID-19 [13]. The overall positive detection rate with NAATs in confirmed cases for sputum samples was estimated by a recent meta-analysis to be 71% (95% CI: 61–80%), which was significantly higher than that of nasopharyngeal and oropharyngeal samples in the same study. More specifically, the estimated percentage of COVID-19-positive samples was 98% (95% CI: 89–100%), 69% (95% CI: 57–80%) and 46% (95% CI: 23–70%) at 0–7 days, 8–14 days and >14 days after symptom onset, respectively [20]. In another meta-analysis, an overall accuracy of 79.7% (95% CI: 43.3–95.3%), sensitivity of 90.1% (95% CI: 83.3–96.9%) and specificity of 63.1% (95% CI: 36.8–89.3%) was estimated for deep-throat saliva/posterior oropharyngeal saliva samples in comparison to nasopharyngeal/oropharyngeal samples in confirmed cases [32].

The infectivity and transmissibility potential of sputum has been demonstrated [21]. If spontaneously produced, sputum collection is an easier process than swab sampling and can easily be done by the patient. The collection of coughed or spit samples carries the potential added risk of transmission by aerosolization [13] (Table 1).

**Table 1:** Characteristics of the alternative specimens/site for detection of severe acute respiratory syndrome coronavirus 2 (SARS-CoV-2).

Site/Fluid	Collection Method ^1^	Self-Sampling	Infectivity Potential	Analyte Detected	Detection Method ^2^	Analyte Load	Approximate Time to Peak	Relative Detection Rate ^3^	Advantage	Disadvantage
Nasopharynx	Synthetic fiber swabs with plastic or wire shafts with	No, healthcare personnel preferred	Yes	Nucleic acid Viral antigen	NAATs IA	High	0–7 days after symptom onset	54% (95% CI: 41–67%) [20]	Gold standard specimen	Needs trained personnel. Procedure is painful and not easy. Not suitable in individuals prone to nose bleeds or has had recent facial or head injury/surgery
Oropharynx	Synthetic fiber swabs with plastic or wire shafts	Possibly, healthcare personnel preferred	Yes	Nucleic acid	NAATs	High	0–7 days after symptom onset	43% (95% CI: 34–52%) [20]	Easy to operate	Less sensitive than nasopharyngeal swab and sputum.
Nasal (mid-turbinate or anterior nares)	Flocked or spun polyester swab	Yes	Yes	Nucleic acid Viral antigen	NAATs IA	Average	0–7 days after symptom onset	82% (95% CI: 73–90%) [22]	Minimally invasive. Lower risk for healthcare infection	Less sensitive if not collected correctly. Not suitable in individuals prone to nose bleeds or has had recent facial or head injury/surgery
Sputum	Sterile container	Yes	Yes	Nucleic acid	NAATs	Average–high	3–7 days after symptom onset	71% (95% CI: 61–80%) [20]	High yield compared to upper respiratory swab	High risk of infection for operators. The high viscosity of sputum makes it difficult to extract nucleic acids.
Saliva	Sterile container	Yes	Yes	Nucleic acid	NAATs	High	3–7 days after symptom onset	>80% [11,20,31]	Not invasive. Less risk for healthcare infection. Large amount of sample	Less sensitive if not collected correctly. False negative results
Broncho-alveolar lavage fluid	Sterile container	No	Yes	Nucleic acid	NAATs	Medium	7–14 days after symptom onset	91.8% (95% CI: 79.9–103.7%) [34]	High detection rate/low limit of detection	High risk of cross-infection
Stool	Stool container	Yes	Not fully clear	Nucleic acid	NAATs	Medium	>14 days after symptom onset	51.8% (95% CI: 43.8–59.7%) [35]	Less risk for healthcare infection. Non-invasive	Might be confined to later-stage infection diagnosis
Urine	Collection tube	Yes	Not fully clear	Nucleic acid	NAATs	Low	16–21 days	5.74% (95% CI: 2.88–9.44%) [36]	Non-invasive sample collection	Limited data has been studied
Ocular fluid	Tear or conjunctival swab	Possibly, healthcare personnel preferred	Not fully clear	Nucleic acid	NAATs	Low	Unclear	0–28.57% [37,38,39,40]	Non-invasive sample collection	No conclusive data available
Blood	Collection tube with anticoagulant	No	Not fully clear	Nucleic acid Antibody	NAATs IA	Low	5–14 days for nucleic acid >10 days for antibody	10% (95% CI: 5–18%) for nucleic acid [41]	Easy to operate, low infectious concern	High false negative rate. High limit of detection.
Serum	Serum separator tubes	No	low	Antibody	IA	High	>14 days for antibody detection	61.2% (95% CI: 53.4–69.0%) for IgM, 58.8% (95% CI: 49.6–68.0%) for IgG, and 62.1% (52.7–71.4%) for IgM-IgG [42]	Rapid, simple and convenient. Sample is more stable. Low cost. Suitable for disease surveillance.	Low sensitivity in the early stage of disease. High false negative rate. Cross-reactivity of antibody and false positive.

^1^ Collected specimen may be stored at room temperature for ≤4 h, at 2–8 °C if ≤72 h, and −70 °C if >72 h. Transport should be on dry ice. ^2^ NAATs: nucleic acid amplification tests. IA: immunoassay. ^3^ Detection rates are relative to other specimens/sites. See the text for detailed description.

### 2.5. Bronchoalveolar Lavage

Bronchoalveolar lavage is generally collected later in the course of COVID-19, from patients with severe illness or undergoing mechanical ventilation or to determine the recovery of admitted patients [13]. A meta-analysis revealed that it has a positive detection rate of 91.8% (95% CI: 79.9–103.7%) by NAATs, which was higher than that of sputum and nasopharyngeal specimen in the same review [34]. Collecting bronchoalveolar lavage is complex and with high risk of aerosolization. It includes the instillation of sterile normal saline into a sub-segment of the lung, followed by suction and collection. Endotracheal aspiration has a lower risk of aerosolization than bronchoalveolar lavage with comparable sensitivity and specificity [43] (Table 1).

### 2.6. Stool

Fecal shedding of respiratory viruses is not uncommon and stool samples may contain large viral loads of SARS-CoV-2 at early onset through the convalescent stage of illness. The World Health Organization (WHO) recommends for stool diagnostic testing to be considered from the second week after symptom onset and onwards, suggesting that this positivity is prolonged compared to that of respiratory tract specimens [13]. Detection rate of SARS-CoV-2 RNA in fecal specimens (excluding anal or rectal swabs) among patients with confirmed diagnosis has been estimated to be 43.7% (95% CI: 32.6−55.0%) in a meta-analysis [44]. This figure was 51.8% (95% CI: 43.8–59.7%) in the most recent meta-analysis which used different inclusion criteria, and also, did not exclude anal or rectal swabs. It was estimated that 64% of tested individuals had persistent positive fecal specimens test despite negative respiratory tests for a mean duration of 12.5 days after negative respiratory testing [35]. Importantly, it has been reported that SARS-CoV-2 viral shedding in stool may persist up to 6 weeks after symptom onset [36]. An overall diagnostic sensitivity of 46.0% (95% CI: 13.1–82.7%) and specificity of 91.4% (95% CI: 6.4–99.9%) has been estimated for feces/anal swab in comparison to nasopharyngeal/oropharyngeal samples in confirmed cases [32].

Fecal–oral transmission is an accepted mode of transmission for other coronaviruses such as SARS-associated coronavirus (SARS-CoV) and Middle East respiratory syndrome coronavirus (MERS-CoV) [45]. Detection of live active SARS-CoV-2 virus in stool samples has been reported in the literature, underlining the possibility of fecal–oral transmission through infected feces. However, it is unclear if the positive fecal test results are due to active virion particles or inactive viral RNA amplified by polymerase chain reaction (PCR). Therefore, the infectivity and transmissibility potential of stool has not been established [21,28,35,45]. Fecal specimens are suggested to be tested concurrently with other samples to detect false-positives and/or monitor disease progression.

Nevertheless, the viral detection rate could vary substantially due to the presence of PCR inhibitors (bile, polysaccharides, hemoglobin and bilirubin) which make the process of viral detection very difficult, susceptible to user error and requiring trained technicians and special RNA extraction kits [17,18,46,47]. Sample collection is simple and can often be performed at home by capturing a stool sample (about 10 g or peanut size) in a dry and clean container and transferring into a sterile specimen cup (Table 1).

### 2.7. Urine

Urinary shedding of SARS-CoV-2 has been highly correlated with disease severity in adults. A recent meta-analysis revealed that the frequency of viral shedding was 4.5% with a weighted pooled estimate of 1.18% (95% CI: 0.14–2.87%) after excluding case reports and case series with small sample size (<9 patients) [48]. The overall urinary shedding of SARS-CoV-2 in confirmed COVID-19 patients was estimated to be 8% in another meta-analysis, with a relative risk of 0.08 (95% CI: 0.05–0.16) compared to nasopharyngeal samples, 0.33 (95% CI: 0.15–0.72) compared to stool samples and 0.20 (95% CI: 0.14–0.29) compared to blood/serum samples [49]. The pooled rate of urine positivity was 5.74% (95% CI: 2.88–9.44%) in another meta-analysis based on different inclusion criteria [36]. However, it remains unclear if urine has infectivity and transmissibility potential despite containing viral genetic material [26,48] (Table 1).

### 2.8. Ocular Fluid

Many respiratory viruses are known to enter through eyes or utilize the eye as a replication site before causing a respiratory infection [50]. SARS-CoV-2 has been detected occasionally in tears and conjunctival swabs in confirmed patients, however, the current data is controversial. The positive detection rate varies greatly in the available studies and figures fluctuate from 0% up to 28.57% [51,52,53,54]. While there are a number of published meta-analysis studies [37,38,39,40], their search dates are not recent and may have omitted newly published studies. A recent meta-analysis reported an overall sensitivity of 17.4% (95% CI: 7.8–34.2%) and specificity of 96.1% (95% CI: 12.7–100%) for ocular fluid in comparison to nasopharyngeal/oropharyngeal samples in confirmed cases [32].

The optimal time window to detect SARS-CoV-2 on the ocular surface, and whether the viral RNA present in the ocular fluids has infectious potential, is still unclear [17,37]. A standardized sample collection method and additional sampling time points would resolve heterogeneity in positive rates and provide insightful information. It has been noted that conjunctiva, cornea or the epithelial cells of the nasolacrimal duct can take up virus and may be a port of entry or direct inoculation site of infectious droplets, leading to contraction of the infection [53]. This is of significant importance for dentists and oral health professionals due the generation of potentially infectious droplets during dental procedures. The collection method includes the use of conjunctival swabs to collect both exfoliated cells and tears, or Schirmer’s test strips to collect tears (Table 1).

### 2.9. Serum, Plasma and Whole Blood

Serum, plasma and whole blood are primarily used in antibody (serology) tests and occasionally for nucleic acid detection for tracking COVID-19 disease progression, severity or prognosis, epidemiological studies and patient immunity [47]. According to the recent meta-analysis of diagnostic performance of serology tests for COVID-19, the pooled sensitivity of Immunoglobin G (IgG), Immunoglobin M (IgM) and combined IgM-IgG tests in confirmed COVID-19 patients was 0.76 (95% CI: 0.65–0.86), 0.69 (95% CI: 0.59–0.78) and 0.78 (95% CI: 0.70–0.85), respectively. Thus, negative serological results alone cannot exclude the diagnosis of COVID-19 [55].

It was further demonstrated that serology tests had the lowest sensitivity at 0–7 days after symptom onset, and the highest sensitivity (more than 85%) at >14 days, suggesting that serological tests might be useful for diagnosis purposes at later stages of disease. The specificity of IgG, IgM and combined IgM-IgG tests was 0.98 (95% CI: 0.96–0.99), 0.95 (95% CI: 0.91–0.98) and 0.97 (95% CI: 0.93–0.99), respectively [55]. In another meta-analysis, SARS-CoV-2 seropositivity rate was estimated to be 61.2% (95% CI: 53.4–69.0%) for IgM, 58.8% (95% CI: 49.6–68.0%) for IgG and 62.1% (52.7–71.4%) for IgM-IgG joint detection in confirmed patients. Serologic testing also yielded high values in the identification of asymptomatic infections with a seropositivity rate of 19% (95% CI: 10.0–27.0%) for combined IgM-IgG [42]. Samples for serology tests are frequently collected by a fingerstick forming the basis for a simple test that can be performed at home by the patient.

SARS-CoV-2 viral RNA has also been detected in blood, serum and plasma samples from patients. A meta-analysis estimated the positive detection rate of viral RNA in blood products up to 28 days following symptomatic onset to be 10% (95% CI: 5–18%) [41], most of which are detected with low copy numbers, at earlier time points and in more severe patients. However, it remains controversial whether the detection of viral RNA in blood samples reflects the presence of infectious virus, as this has important safety implications, especially for dental practitioners and personnel and those handling patient-related materials in clinical, laboratory and research environments [41]. For RNA detection, 5 mL of anticoagulated blood is required. Vacuum tubes containing ethylenediaminetetraacetic acid (EDTA) anticoagulant are recommended for blood collection (Table 1).

## 3. Discussion

Recommended infection prevention and control practices for dental treatment delivery encourage the elective procedures and non-urgent outpatient visits to be postponed in applicable circumstances, and tele-dentistry and triage protocols to be implemented prior to dental appointments. The next step is to screen and triage everyone entering the dental office for fever and symptoms consistent with COVID-19 or exposure to others with COVID-19 infection. Nevertheless, a fever might only be associated with a dental diagnosis if no other symptoms of COVID-19 are present. The patients should also be requested to contact the dental office if they develop COVID-19 signs or symptoms or are diagnosed with COVID-19 within 2 days after the dental appointment [3,56]. However, pre-symptomatic (before symptom onset), or asymptomatic patients (that account for more than 40% of confirmed cases), impose a greater challenge than symptomatic patients for dental settings in this process [57]. The high transmissibility of SARS-CoV-2 has been attributed to asymptomatic carriers and pre-symptomatic patients. These patients have similar viral load to that of symptomatic COVID-19 patients, causing comparable transmissibility [58].

There are very limited studies which reported the prevalence of COVID-19 infection among dental patients. Lamberghini et al. [59] reported an overall SARS-CoV-2 positivity rate of 2.3% in asymptomatic children attending a high-volume pediatric dental practice. Conway et al. [60] reported an overall test positivity rate of 0.6% (95% CI: 0.4–0.8%) in child and adult asymptomatic patients attending multiple dental care centers. These findings highlight that while dental practices must screen patients for signs and symptoms of COVID-19 and refer patients for appropriate medical follow-up when indicated, such screening alone will not identify all individuals who are infected. Therefore, timely, accurate (highly sensitive and specific) and rapid screening and diagnostic testing that can distinguish COVID-19 cases from healthy or other virus-infected individuals is an essential need to take required actions, optimize patient care, maintain dental patients’ and treatment providers’ safety and to contain and prevent disease spread. It is being recognized that dental practices would greatly benefit from the ability to evaluate the disease status of their patients by using point-of-care COVID-19 diagnostic tests.

## 4. Conclusions

SARS-CoV-2 can be detected in different body specimens and sites. Dentists, directly or indirectly, deal with many of these specimens/sites in their daily practices. Information regarding prevalence of SARS-CoV-2 virus among asymptomatic individuals is less well-documented but is significant in future management of the dental environment. The present literature indicates that detection of SARS-CoV-2 and the infectious potential of the tested virus is dependent on the time of specimen collection relative to symptoms/infection and the presence of actual live viral particles.

## Data Availability

No new data were created or analyzed in this study. Data sharing is not applicable to this article.
